# Geochemical characterization and health risk assessment of groundwater in Wadi Ranyah, Saudi Arabia, using statistical and GIS-based models

**DOI:** 10.1007/s10653-025-02517-6

**Published:** 2025-05-16

**Authors:** Ahmed A. Asmoay, Eltaher M. Shams, Wael F. Galal, Ahmed Mohamed, Rashad Sawires

**Affiliations:** 1 Geological Science Department, National Research Centre, Advanced Materials Technology and Mineral Resources Research Institute, Al-Behoos St., Dokki, Cairo, 12622 Egypt; 2 Natural Resources and Energy Department, Damanhur University, Damanhur, 22511 Beheira Egypt; 3 Department of Geology, Faculty of Science, Assiut University, Assiut, 71516 Egypt

**Keywords:** Groundwater contamination, Heavy metals, Hydrochemical classification, Health risk assessment, Wadi Ranyah, Saudi Arabia

## Abstract

Groundwater in Wadi Ranyah, the main water source for local communities, was analyzed using 77 samples to evaluate physicochemical properties, major ions, and heavy metal concentrations. While most parameters met World Health Organization (WHO) standards, levels of arsenic, lead, cadmium, chromium, and nickel exceeded permissible limits. Hydrochemical analyses were conducted using Piper and Durov diagrams, alongside health risk assessments based on statistical ratios established by the United States Environmental Protection Agency (US EPA). The analysis identified two dominant water types (SO_4_·Cl–Ca·Mg and HCO_3_–Ca·Mg), influenced by ion exchange, evaporite dissolution, and silicate weathering. Health risk assessment, based on US EPA models, revealed significant non-carcinogenic and carcinogenic risks, particularly for children. Oral ingestion accounted for the majority of exposure, with arsenic and lead being the most hazardous. Dermal exposure risks were comparatively lower. The identified health threats include potential dermatological, cardiovascular, and neurological effects, and an increased cancer risk. Based on these findings, groundwater in Wadi Ranyah is unsuitable for drinking without treatment. Mitigation strategies such as reverse osmosis, ion exchange filtration, and continuous monitoring are recommended to reduce heavy metal contamination and protect public health.

## Introduction

Groundwater contamination by heavy metals is a significant health risk globally, particularly in regions where industrial activities, agricultural practices, and inadequate waste management systems prevail. Heavy metals like lead, cadmium, chromium, and mercury are of particular concern due to their toxicological effects and long-lasting presence in ecosystems. These pollutants infiltrate groundwater systems through various pathways, including industrial effluent discharge, agricultural runoff (fertilizers and pesticides), and improper waste disposal (e.g., Singh et al., 2017; Karami et al., 2018; Mallick et al., 2018; Farrag et al., 2019; Kumar, 2019; Sharmin et al., 2020; Zaghlool, 2020; Lee et al., 2021; Arifullah et al., 2022; Eslami et al., 2022; Ghani et al., 2022; Eziz et al., 2023; Ullah et al., 2023; Radouane et al., 2021; Asmoay et al., 2024; Eid et al., 2024; Karadeniz et al., 2024; Benyoussef et al., 2024; Asmoay, 2025; Chowdhury et al., 2025). As these pollutants persist in the environment, they pose long-term threats to human health, needing effective monitoring and management.

In Saudi Arabia, the contamination of groundwater by heavy metals has emerged as a major environmental issue, primarily due to the country’s reliance on groundwater as a vital resource for drinking and irrigation in its arid climate (Alhagri et al., 2024). Heavy metals such as arsenic (As), lead (Pb), cadmium (Cd), chromium (Cr), and mercury (Hg) are particularly disturbing due to their toxic properties and potential bioaccumulation within the food chain (e.g., Ben-tahar et al., 2025a; Mahjoub et al., 2024). Groundwater degradation in the country has been linked to anthropogenic activities, including industrial waste discharge, agricultural runoff, and inadequate waste management practices, all of which threaten public health (Ali et al., 2020).

Recent studies in Saudi Arabia have revealed alarming concentrations of heavy metals in groundwater (e.g., Alhagri et al., 2024; Alharbi & El-Sorogy, 2024; Ali et al., 2020; Jibrin et al., 2024; Khan et al., 2023). For instance, in the Al-Qassim region, heavy metal levels were found to exceed World Health Organization (WHO) and Gulf Standard Organization guidelines, particularly in areas associated with agricultural activities and industrial waste (Alharbi & El-Sorogy, 2024). In Bukayriyah city, despite average concentrations being within safe limits, groundwater in localized regions presented significant health risk**,** especially for vulnerable groups such as children and pregnant women (Alhagri et al., 2024). These findings underscore the importance of assessing and mitigating groundwater contamination in the region.

Health risk assessments are crucial for evaluating the impacts of heavy metal exposure from contaminated groundwater. Commonly used indices, such as Chronic Daily Intake (CDI), Hazard Quotient (HQ), and Total Carcinogenic Risk (TCR), help quantify these risks (e.g., Alhagri et al., 2024; Alharbi & El-Sorogy, 2024; Asmoay, 2025; Asmoay et al., 2023, 2024; Manawi et al., 2024; Salman et al., 2019). For instance, research in the Khulais region demonstrated that heavy metals like arsenic and chromium posed significant carcinogenic risk, emphasizing the need for intervention strategies (Khan et al., 2023). Similarly, studies in Qatar and Morocco revealed similar risk, particularly for children, due to high concentrations of heavy metals linked to agricultural practices (Manawi et al., 2024; Sanad et al., 2024). In Egypt, investigations have integrated simulations with conventional risk assessment approaches to better understand the carcinogenic risk posed by heavy metal contamination in groundwater sources (Asmoay et al., 2023, 2024). Such studies demonstrated the widespread nature of this issue and the urgent need for effective mitigation.

The evolution of health risk assessment techniques has enhanced our understanding of groundwater quality issues. Innovative approaches, such as machine learning algorithms, have been employed to forecast water quality indices and manage complex hydrogeological data in regions such as the Eastern Province of Saudi Arabia (Jibrin et al., 2024). These methods offer valuable tools for improving groundwater monitoring and management strategies. Given the significance of groundwater as a resource in Saudi Arabia, ensuring its safety from heavy metal contamination is critical for public health and sustainable water management.

Wadi Ranyah, a key region in Saudi Arabia, is heavily dependent on groundwater for agricultural and domestic use. However, this area faces significant challenges from heavy metal pollution, making the assessment of water quality and health risks essential. This research aims to address these challenges by evaluating the suitability of water for consumption, identifying processes governing water chemistry, classifying water types, assessing the presence and risk of heavy metals, and projecting the future of groundwater in the region. Notably, our work introduces a novel integration of geochemical analysis, health risk assessment, and the application of statistical and GIS-based models, specifically tailored to the Wadi Ranyah region. We have also provided a comprehensive evaluation of health risk posed to human populations, a focus that, to the best of our knowledge, has not been explored in this region despite similar studies being conducted elsewhere.

## Geologic and hydrogeologic settings

**Fig. 1:**
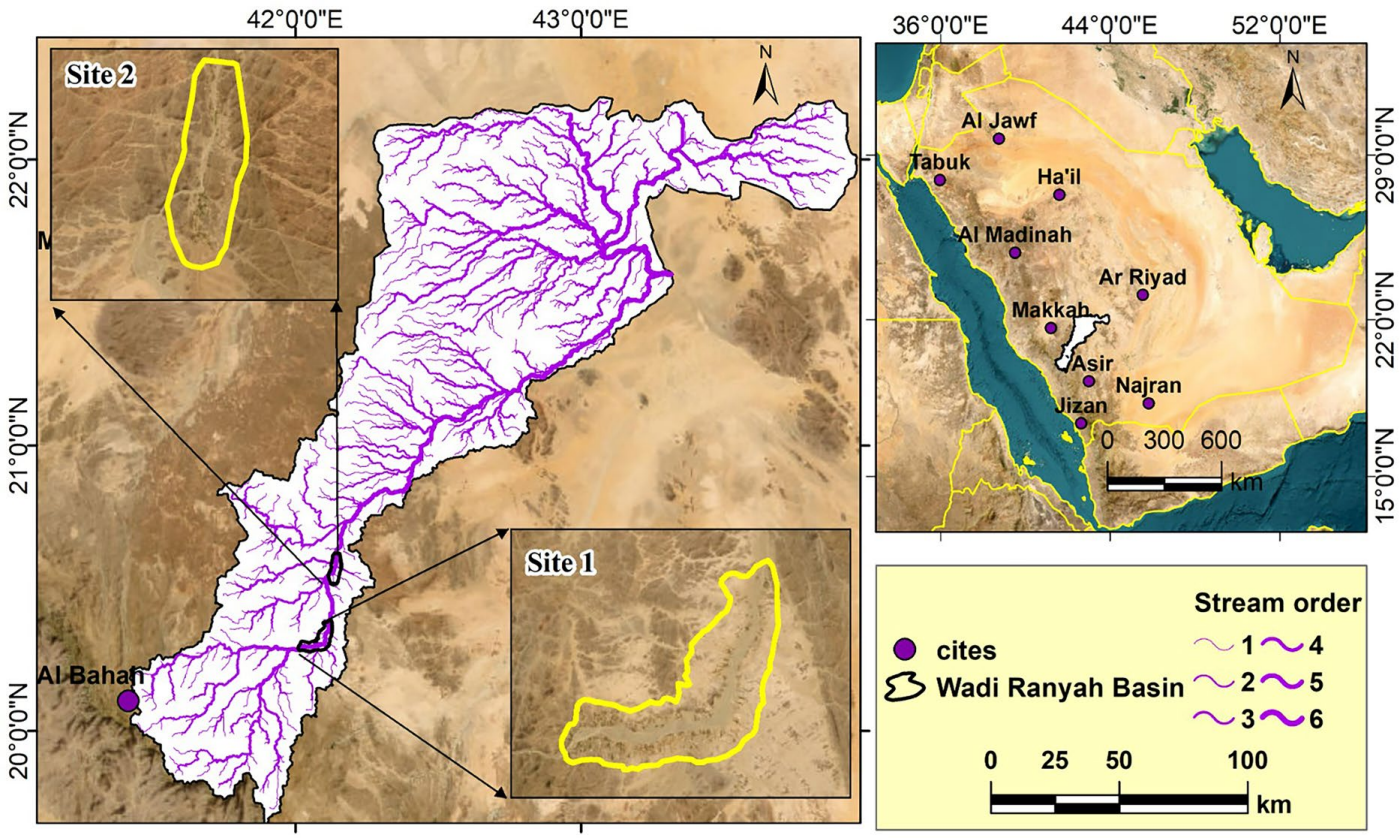
Location map of the Wadi Ranyah Basin, Saudi Arabia, illustrating the stream orders within the valley and the studied groundwater sampling sites

Western Saudi Arabia experiences significant rainfall, contributing to valuable groundwater reserves within wadis and streams (Şen, 1983). Wadi Ranyah, one of the primary groundwater sources in the Al-Baha region, has its origin in the highlands of Al-Baha and flows northeast, ultimately reaching the desert beyond Ranyah city (Fig. 1). Covering an area of approximately 2500 km^2^, Wadi Ranyah is a prominent geological feature situated within the Arabian Shield of southwestern Saudi Arabia. This endorheic wadi, extending about 245 km in length, ranks among the longest in the region, beginning in the Baha Mountains and stretching towards the northeastern desert plains (Saleem et al., 2020).

**Fig. 2:**
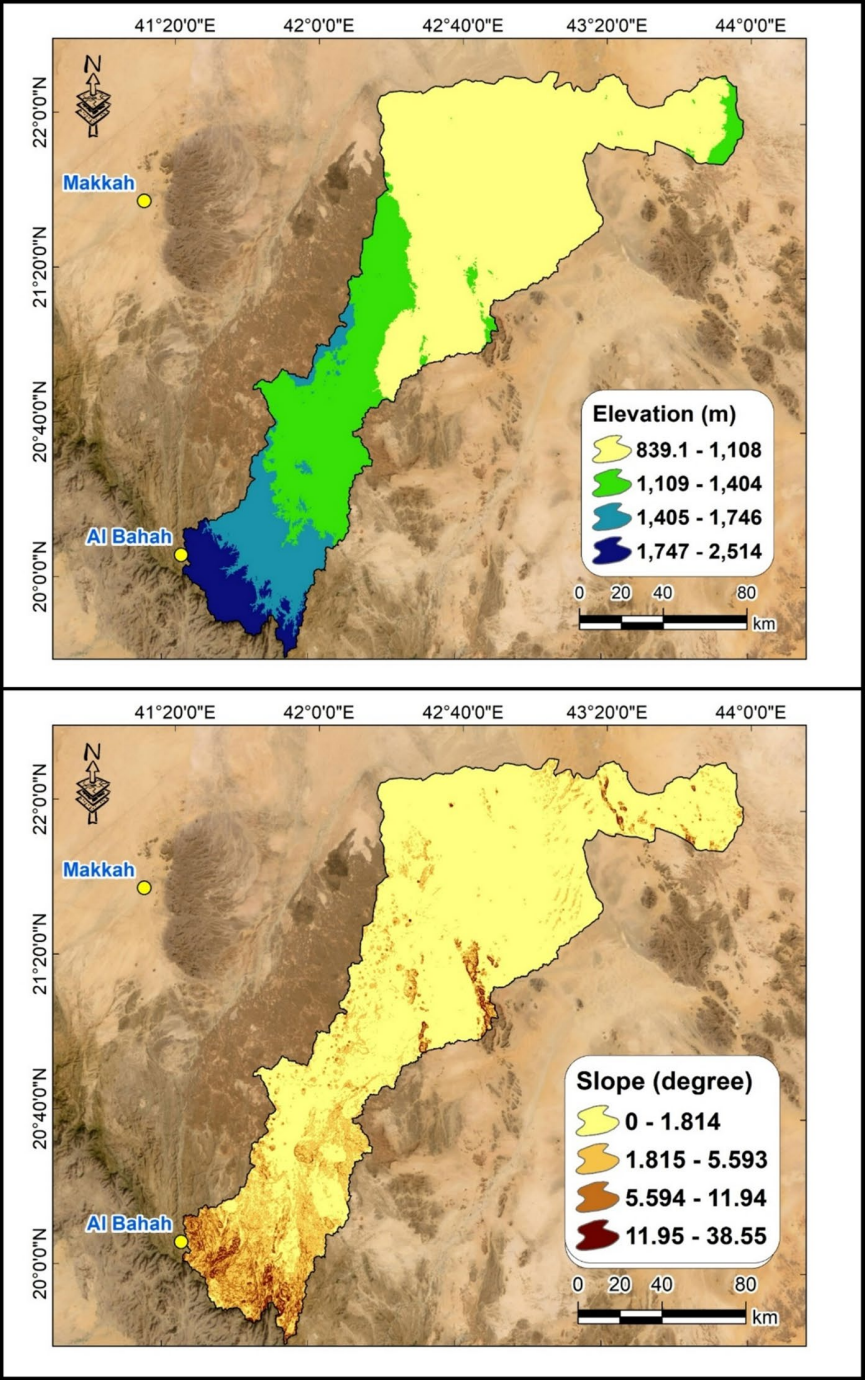
Upper plot represents the elevation profile along Wadi Ranyah Basin, and the lower one illustrates the estimated slopes along the same basin

The topography of Wadi Ranyah shows a significant variation in elevation from its upstream origin in the Baha Mountains to its downstream extent. In the upstream regions, elevations range between 1750 and 1880 m above mean sea level (amsl), particularly in the high, well-defined stream areas. As the wadi flows northeastward, the terrain slopes downward, and the elevation gradually decreases to approximately 850 m amsl in the downstream areas near the desert (Fig. 2). The stream network of Wadi Ranyah begins with a steep gradient in the mountainous regions of Baha, providing the necessary energy for surface runoff and erosion. Moving downstream, the slope becomes progressively gentler, resulting in slower water flow and influencing sediment deposition along the wadi’s course. This topographic gradient plays a crucial role in the hydrological and sedimentary dynamics of the area, shaping both the wadi’s landscape and its groundwater recharge potential (Saleem et al., 2020).

**Fig. 3:**
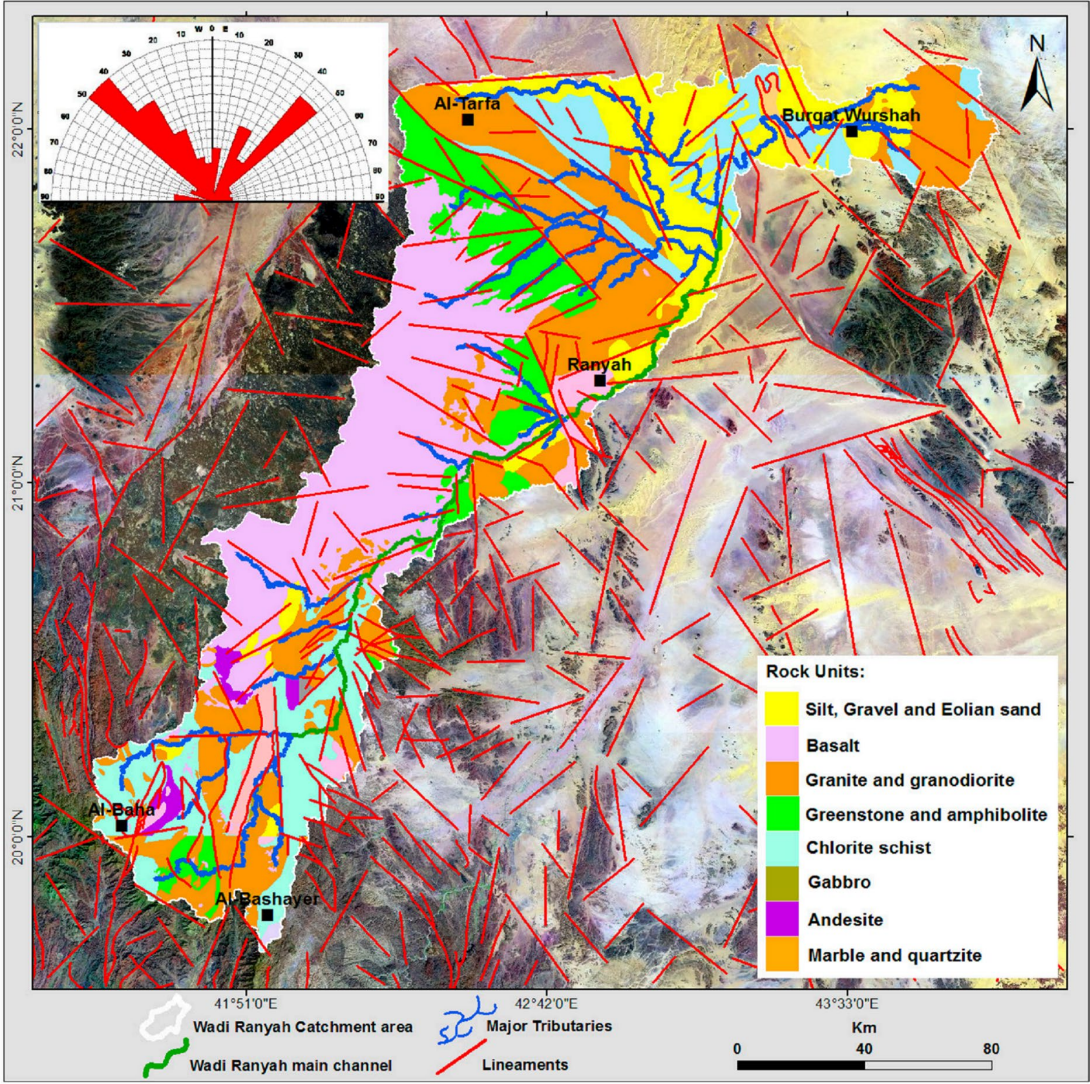
Map depicting the geological features of the study area (after Mohamed et al., 2023)

Geologically, Western Saudi Arabia is characterized by diverse formations ranging from the Precambrian to the Quaternary period (Fig. 3). The region includes the Precambrian rocks of the Baha Group, as well as plutonic and hypabyssal intrusive rocks, and more recent Quaternary alluvial deposits. The Jeddah Group, part of this geological framework, comprises metavolcanic and metasedimentary rocks, such as andesitic flows. The intrusive rocks feature a variety of lithologies, including mafic dikes, granites, tonalite gneiss, and diorites (Saleem et al., 2020; Simons, 1988).

The geology of Wadi Ranyah specifically is marked by an extensive exposure of rock units including metamorphic basalt, andesite, basaltic flows, pyroclastic dacite, rhyolite, and metamorphosed volcanic wacke and sandstone (Fig. 3). Additionally, plutonic rocks such as granodiorites, diorites, granitic plutons, gneiss, and schists are present, contributing to the region’s tectonic and mineralogical diversity. Quaternary alluvial deposits in the wadi consist of layers of gravel, eolian sands, silts, and clayey sands. These sediments, eroded from surrounding basement exposures, range in thickness from 5 to 10 m upstream to 10–15 m or more downstream, forming a highly permeable substrate. This permeability influences both surface runoff and the potential for groundwater storage (Saleem et al., 2020).

The climate of the study area is characterized by cold winters and warm summers, with significant seasonal variations in temperature and rainfall. Evaporation rates are notably high throughout the year. In the upstream region of Wadi Ranyah, the mean annual rainfall is approximately 470 mm (Mohamed et al., 2025). A considerable portion of this rainfall contributes to the surface runoff, which is most common during the winter and spring seasons. These runoff events are crucial for recharging groundwater, shaping the hydrological dynamics of the region.

Hydrogeologically, the Wadi Ranyah aquifer is primarily composed of alluvial deposits along the wadi’s course, with thicknesses varying from approximately 3 m in the upstream areas to around 12 m downstream. These alluvial sediments, consisting of sands, gravels, silts, and clays, contribute significantly to groundwater storage. Additionally, fractured bedrock formations in the region serve as secondary aquifers, enhancing water retention and movement. The water table depth in the Wadi Ranyah aquifer fluctuates between 3 and 10 m, but this variability does not follow a consistent pattern along the wadi’s length. This irregularity may be attributed to local geological and hydrological conditions, including variations in recharge, subsurface structure, and permeability of the aquifer materials (Subyani & Al Ahmadi, 2010; Mohamed et al., 2023). In areas where highly weathered and fractured hard rocks are exposed along the wadi floor and its tributaries, conditions are favorable for shallow groundwater storage. Structurally, the region is affected by faults and fractures, which act as channels for groundwater flow and reservoirs for storage, underscoring the hydrogeological significance of Wadi Ranyah.

## Materials and methods

### Collection and preparation of groundwater samples

**Fig. 4:**
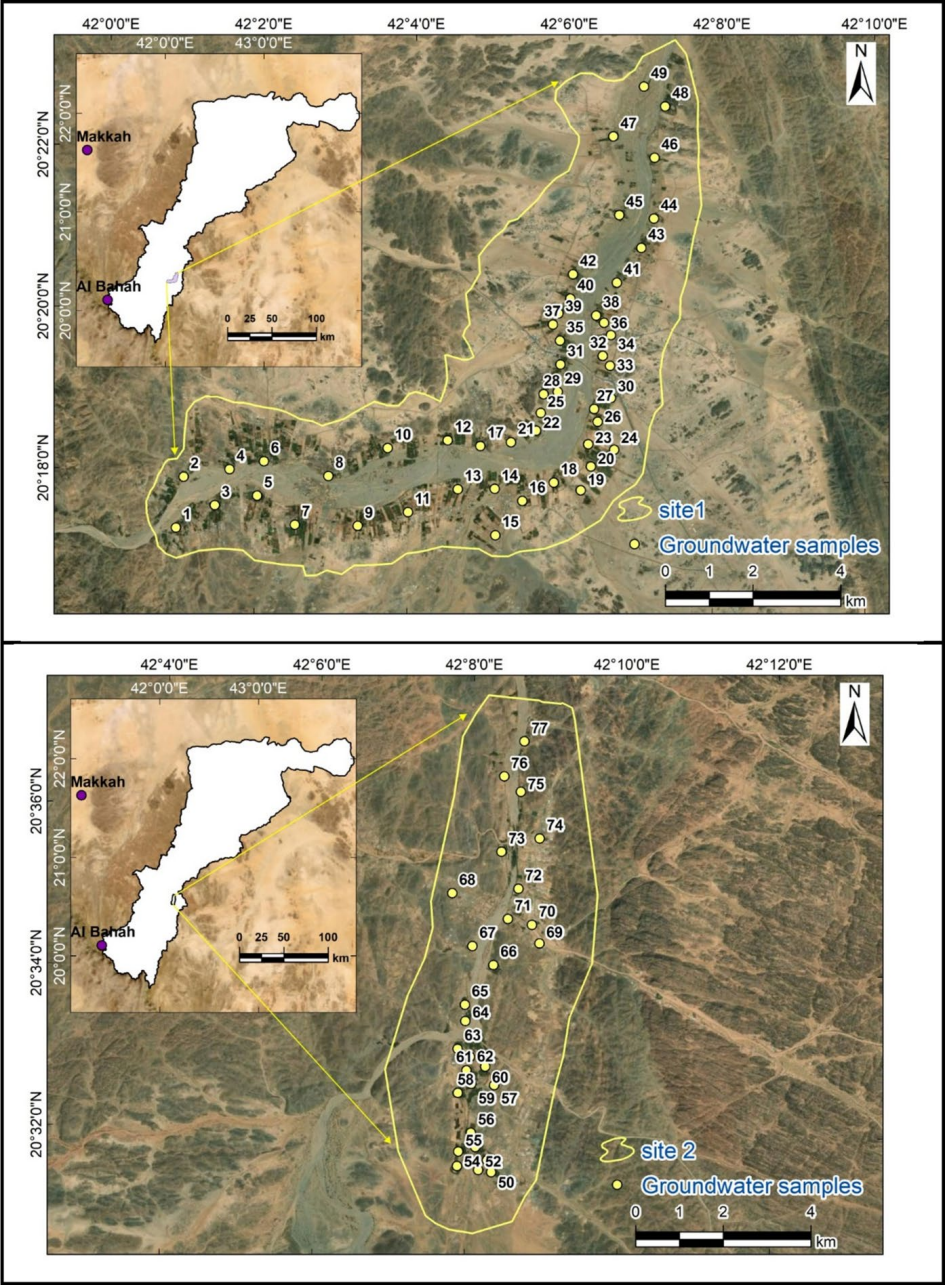
Locations of the analyzed groundwater samples at both study sites in Wadi Ranyah, with Site 1 shown in the upper plot and Site 2 in the lower plot

Seventy-seven groundwater samples (Mohamed et al., 2025) were collected from Wadi Ranyah, Saudi Arabia, in June 2024 (see Fig. 4 & Appendix 1). These groundwater samples were gathered in one-liter containers. The containers were subjected to a cleaning process with a 1:1 diluted nitric acid solution and subsequently rinsed with distilled water. In accordance with standard procedures (APHA, 2017), containers were used to collect water samples, which were first thoroughly washed with the groundwater sample itself to prevent contamination and then prepared in situ by rinsing them once more with the same sample. Water samples were collected and promptly analyzed using portable meters (Hanna USA H-198130) for measurements of pH, electrical conductivity (EC), and total dissolved solids (TDS). Principal ions, including anions (bicarbonate “HCO_3_^−^”, chloride “Cl^−^”, sulfate “SO_4_^2−^”, and nitrate “NO_3_^−^”) and cations (calcium “Ca^2+^”, magnesium “Mg^2+^”, sodium “Na^+^”, and potassium “K^+^”), were assessed following the established protocol (APHA, 2017), as detailed in Table (1). The titration technique was employed to quantify calcium, total hardness (TH), chloride “Cl^−^”, bicarbonate “HCO_3_^−^”, and phosphate “PO_4_^3−^” levels. Potassium “K^+^” and sodium “Na^+^” concentrations were determined through flame photometry, while a spectrophotometer was utilized to analyze the sulfate “SO_4_^2−^” and nitrate “NO_3_^−^” concentrations. Atomic absorption spectrometry (AAS) was employed to ascertain the levels of silica “SiO_2_” and heavy metals (Nickel “Ni”, Cadmium “Cd”, Lead “Pb”, Chromium “Cr”, and Arsenic “As”). The sum of cations and anions was utilized for computing the analytical error, which was established to be ± 5% (Tables 1 and 2), thereby reflecting the precision of the chemical data (Asmoay et al., 2024).

**Table 1:** Standardized methods for assessing variables in the groundwater of the study area

Parameter	Method/instrument	Reagents
pH	pH-meter (Hanna USA H-198130)	Potassium Chloride (KCl)
EC (μS/cm)		
TDS (ppm)		
TH as CaCO_3_ (ppm)	Titrimetric	Hydrochloric Acid (HCl) and Standard EDTA solution
Ca^2+^ (ppm)	Titrimetric with EDTA	EDTA, Sodium hydroxide (NaOH) and Murexide
Mg^2+^ (ppm)	TH-Ca	Based on estimations
Na^+^ (ppm)	Flame photometer (Elico) (Systronics, 128)	Sodium Chloride (NaCl), KCl and Calcium Carbonate (CaCO_3_)
K^+^ (ppm)		
HCO_3_^−^ (ppm)	Titrimetric	Hydrosulfuric Acid (H_2_SO_4_), Methyl Orange
Cl^−^ (ppm)		Silver Nitrate, Potassium Chromate
SO_4_^2−^ (ppm)	UV–Visible spectrophotometer (Spectronic 21, BAUSCH and LOMB)	Glycerol, HCl, Ethyl Alcohol, NaCl, BaCl_2_, Sodium Sulphate
NO_3_^−^ (ppm)		Brucine-Sulpanilic Acid, KNO_3_ and H_2_SO_4_
PO_4_^3−^ (ppm)	Titrimetric	Following APHA (2017) standard protocol
SiO_2_ and heavy metals (Ni, Cd, Pb, Cr & As) (ppm)	Atomic absorption Spectrometry (AAS, PerkinElmer 400)	Following APHA (2017) standard protocol

**Table 2:** US EPA guidelines for different coefficients

Parameter	Significations	Children	Adults
R*f*D Oral (mg.kg^−1^ d^−1^) for Ni, Cd, Pb, Cr and As	Reference dose of a particular non-carcinogenic substance in water	0.02, 0.0005, 0.0014, 0.003, and 0.0003, respectively	0.02, 0.0005, 0.0014, 0.003, and 0.0003, respectively
R*f*D Dermal (mg.kg^−1^ d^−1^) for Ni, Cd, Pb, Cr and As		5.4 × 10^–3^, 5 × 10^–6^, 4.2 × 10^–4^, 1.5 × 10^–5^, and 1.23 × 10^–4^, respectively	5.4 × 10^–3^, 5 × 10^–6^, 4.2 × 10^–4^, 1.5 × 10^–5^, and 1.23 × 10^–4^, respectively
CSF for Ni, Cd, Pb, Cr and As	Cancer slope factor	0.91, 15, 0.0085, 0.42, and 1.5, respectively	0.91, 15, 0.0085, 0.42, and 1.5, respectively
IR (L.d^−1^)	Rate of water consumption	1.2	2
EF (d.a^−1^)	Frequency of exposure	365	365
ED (a)	Duration of exposure	6	30
BW (kg)	Weight of residents	28	65
AT for non-carsinogenic (d)	Life expectancy of residents	2190	10950
AT for carinogenic (d)	Life expectancy of residents	4380	25550
SA (cm^2^)	Skin contact surface area	6.6 × 10^3^	1.8 × 10^4^
K_P_ (cm.h^−1^)	Skin permeability coefficient	0.001	0.001
EV	Frequency of bathing	1	1
ET (h.d^−1^)	Bath duration	1	0.58
CF (L.cm^−2^)	Volume conversion factor	0.001	0.001

### Spatial distribution, statistical analysis, and hydrochemical modeling

**Fig. 5:**
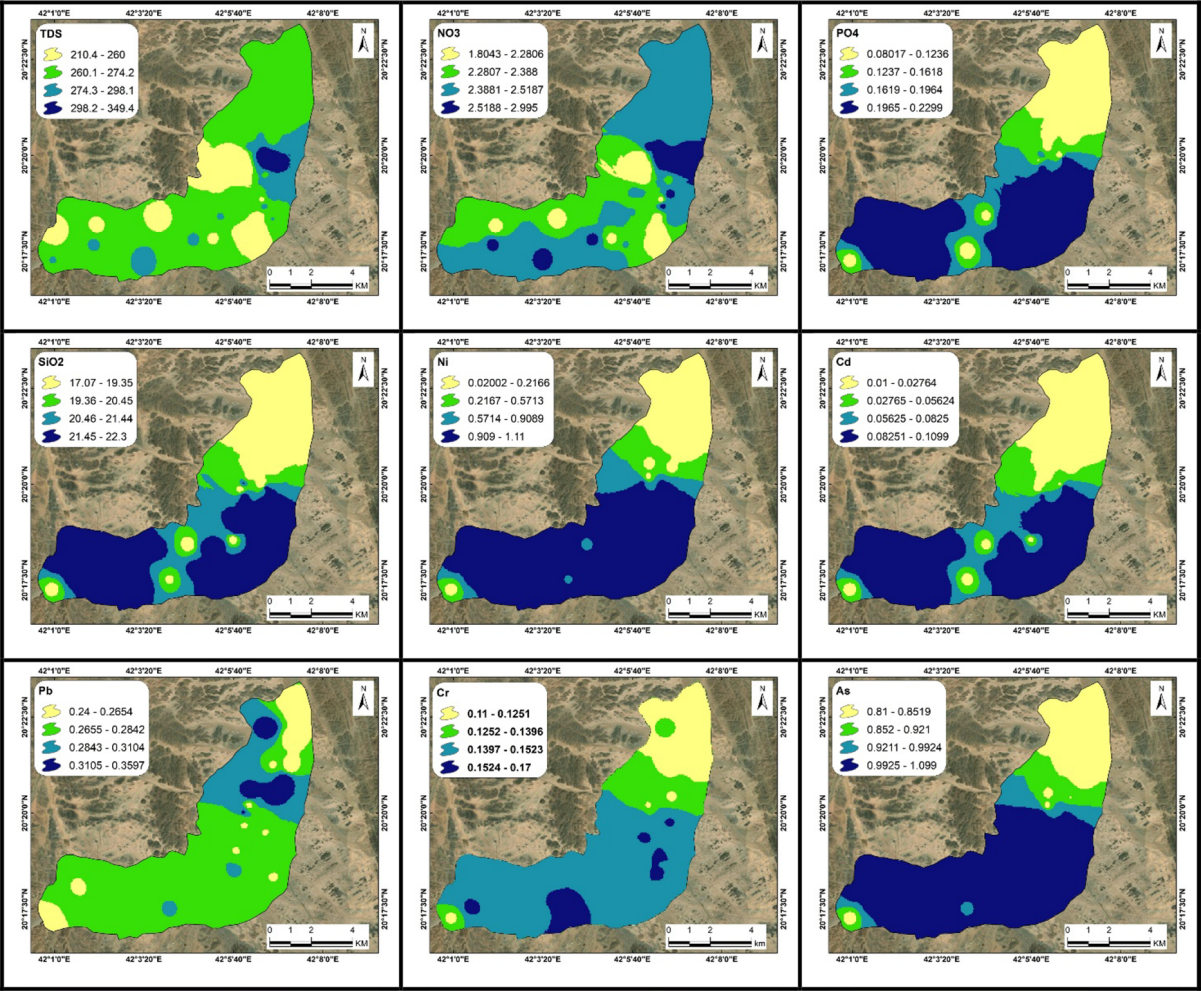
Distribution maps of TDS, NO_3_, PO_4_, SiO_2_, Ni, Cd, Pb, Cr, and As (ppm) in groundwater samples in the site number 1. Additional parameters are presented in Appendix 2

**Fig. 6:**
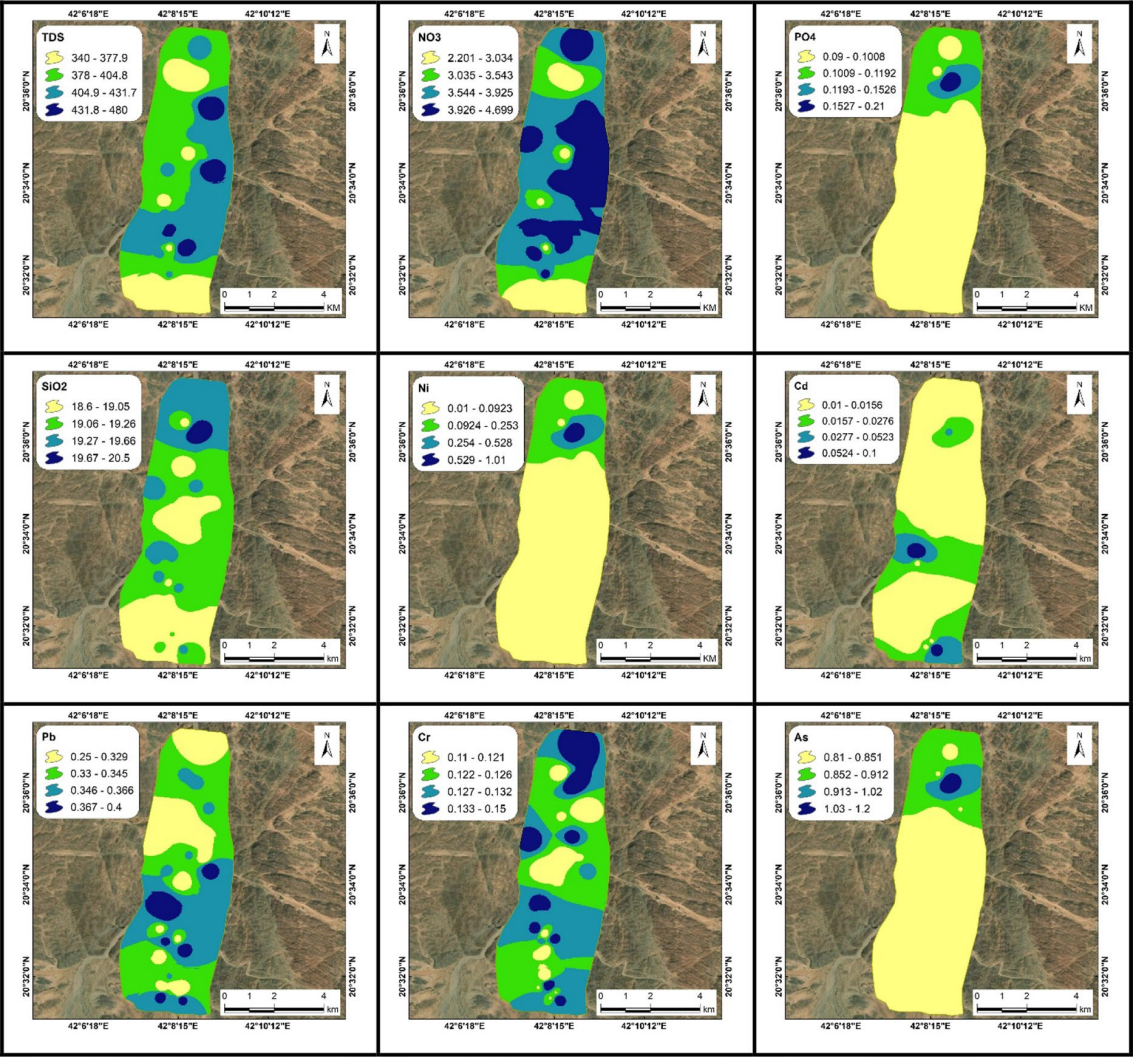
Distribution maps of TDS, NO_3_, PO_4_, SiO_2_, Ni, Cd, Pb, Cr, and As (ppm) in groundwater samples in the site number 2. Additional parameters are presented in Appendix 3

Following the measurement of anions, cations, and heavy metals concentrations, the data were input into ArcGIS 10.8 software (Esri, Berkeley, CA, USA) to generate zoning maps for the two studied sites along Wadi Ranyah (Figs. 5, 6 and Table 3 and Appendices 2, 3). These maps were developed and analyzed according to WHO standards (WHO, 2022), with groundwater quality classified based on the permissible limits for each parameter. Concentrations exceeding these limits are deemed unsafe for consumption, while those within the limits are considered safe. To visualize the spatial distribution of the studied elements in the GIS software, the inverse distance weighted (IDW) model was applied. This method was utilized to zone all concentrations across the study area.

**Table 3:** Statistical parameters (minimum, maximum, mean, median, and quartiles) for the groundwater samples, in comparison to WHO (2022) guidelines

Variables	Min	Max	Mean	Median	Quartile			WHO (2022)
					Q1	Q2	Q3	
T (°C)	19	28	23	23	22	23	24	
pH	6.8	7.5	7.0	7.3	7.2	7.3	7.3	6.5–8.5
EC (µS/cm)	382	873	571	504	482	504	636	1500
TDS (ppm)	210	480	314	277	265	277	350	1000
TH (ppm)	113	187	150	149	140	149	156	500
Ca^2+^ (ppm)	25	52	40	40	37	40	42	75
Mg^2+^ (ppm)	11	15	12	12	12	12	12	100
Na^+^ (ppm)	22	35	28	28	27	28	29	250
K^+^ (ppm)	1	2	1	1	1	1	2	12
HCO_3_^−^ (ppm)	120	185	137	128	126	128	131	250
Cl^−^ (ppm)	38	55	47	48	45	48	50	250
SO_4_^2−^ (ppm)	29	50	39	40	38	40	42	250
NO_3_^−^ (ppm)	1.8	4.7	2.8	2.5	2.4	2.5	2.8	50
PO_4_^3−^ (ppm)	0.1	0.2	0.2	0.1	0.1	0.1	0.2	1
SiO_2_ (ppm)	17	22	20	19	19	19	22	24
Ni (ppm)	0.0	1.1	0.5	0.9	0.0	0.9	1.1	0.07
Cd (ppm)	0.0	0.1	0.1	0.0	0.0	0.0	0.1	0.003
Pb (ppm)	0.2	0.4	0.3	0.3	0.3	0.3	0.4	0.01
Cr (ppm)	0.1	0.2	0.1	0.1	0.1	0.1	0.2	0.05
As (ppm)	0.8	1.2	0.9	1.0	0.8	1.0	1.0	0.01

In addition, statistical analyses and graphical representations for the studied elements and sites were conducted using Microsoft Excel 365 and Grapher 16.2.354 software. Descriptive statistical analyses were performed to evaluate the distribution patterns and variability of variable concentrations concerning WHO standards. The calculations included the maximum and minimum values, mean, and median, as well as quartiles (Q1, Q2, and Q3), providing insights into data dispersion and central tendency. These statistical measures facilitated a comprehensive understanding of the concentration trends and their compliance with international health guidelines.

Piper’s and Durov’s diagrams were also formulated with the assistance of AqQA LLC. 1.1.5.1. The Piper diagram is a widely used graphical tool for analyzing and classifying hydrochemical data in groundwater and surface water studies. Introduced by Piper (1944), this ternary plot consists of two lower triangular fields representing the relative proportions of major cations (Ca^2+^, Mg^2+^, Na⁺ + K⁺) and anions (HCO₃⁻, SO₄^2−^, Cl⁻), which are projected onto a central diamond-shaped field to visualize overall water types and hydrochemical facies. This approach facilitates the identification of geochemical evolution, mixing processes, and water–rock interactions. The Piper diagram is particularly useful in hydrogeological investigations, water quality assessments, and environmental studies, providing a clear and systematic way to interpret complex water chemistry datasets.

On the other hand, the Durov diagram is a graphical representation used in hydrochemical studies to classify water types and interpret geochemical processes. Developed by Durov (1948), this plot consists of two ternary diagrams for major cations (Ca^2^⁺, Mg^2^⁺, Na⁺ + K⁺) and anions (HCO₃⁻, SO₄^2^⁻, Cl⁻), which are projected onto a square field. The arrangement of water samples within the diagram helps identify hydrochemical facies, mixing trends, and processes such as ion exchange, dissolution, and water–rock interaction. Compared to the Piper diagram, the Durov diagram provides additional insights into hydrochemical evolution by incorporating a subdivision of water types based on their ionic composition. It is widely used in groundwater quality assessment, geochemical modeling, and environmental monitoring.

Similarly, the Gibbs diagram is a widely used graphical tool for understanding the geochemical controls on water chemistry, particularly in natural water systems. Introduced by Gibbs (1967), this diagram plots the weight ratio of major anions (Cl⁻/(Cl⁻ + HCO_3_⁻)) or cations (Na⁺/(Na⁺ + Ca^2^⁺)) against total dissolved solids (TDS) to identify the dominant processes influencing water composition. These processes are categorized into three main mechanisms: precipitation dominance, rock dominance, and evaporation dominance. The Gibbs diagram is particularly useful in distinguishing between water chemistry influenced by atmospheric precipitation, water–rock interaction, and evaporative concentration, making it a valuable tool in hydrogeological and environmental studies.

The integration of GIS-based spatial analysis, statistical evaluations, and hydrochemical modeling ensured a comprehensive understanding of element distribution, potential risks, and influencing factors across the study area.

### Health risk assessment of the heavy metals content

The statistical methodology applied in this study provides a robust framework for evaluating the health risk associated with hazardous water contaminants. This approach involves a comprehensive process, including hazard identification, dose–response assessment, exposure analysis, and risk characterization (Asmoay et al., 2023, 2024; Salman et al., 2019). Specifically, this research focuses on the health risk posed by heavy metals in groundwater, employing the well-established risk assessment framework recommended by the US EPA (2011). To quantify this risk, the study utilizes a set of equations (Eqs. 1, 2, 3, 4, 5 and 6), as outlined by previous works in the field (Alhagri et al., 2024; Alharbi & El-Sorogy, 2024; Ali et al., 2020; Khan et al., 2023), ensuring accurate and reliable risk assessments.

1 $$HI = HQ_{{Oral}} {\text{ }} + HQ_{{Dermal}} {\text{ }}$$

2 $$TCR=\sum_{i=1}^{n}CR$$

The US EPA (2011) guidelines have established a threshold of unity for both the non-carcinogenic hazard quotient (HQ) and hazard index (HI). Similarly, the total cancer risk (TCR) and individual cancer risk (CR) are defined with a maximum permissible threshold of 10⁻^6^.

3 $$HQ=\frac{\text{CDI}}{{\text{RfD}}}$$

4 $$CR = CDI \cdot CSF$$

CDI (mg·kg⁻^1^·d⁻^1^) represents the daily intake of contaminants, which is crucial for evaluating both non-carcinogenic and carcinogenic health risks. RfD (mg·kg⁻^1^·d⁻^1^) refers to the reference dose for a specific non-carcinogenic substance present in water, while CSF denotes the cancer slope factor associated with each heavy metal. The primary exposure pathways for heavy metals in contaminated water are through ingestion of water with elevated metal concentrations and dermal absorption from contact with polluted water (US EPA, 2014; Ali et al., 2020; Khan et al., 2023; Alharbi & El-Sorogy, 2024; Alhagri et al., 2024).

5 $${\text{CDI }}_{\text{Oral}}=\frac{C. IR. EF. ED}{BW. AT}$$

6 $${\text{CDI }}_{\text{Dermal}}=\frac{C. SA. {\text{K}}_{\text{P }}. EV. ET. EF. ED. CF}{BW. AT}$$

The definition and scope of the various parameters used in analyzing the groundwater quality provide a comprehensive understanding of the non-carcinogenic and carcinogenic risks associated with five heavy metals: Ni, Cd, Pb, Cr, and As (Tables 4, 5; US EPA, 2014; Ali et al., 2020; Khan et al., 2023; Alharbi & El-Sorogy, 2024; Alhagri et al., 2024).

**Table 4:** Ratios of non-carcinogenic effects (*CDI* Chronic Daily Intake, *HQ* Hazard Quotient, *HI* Hazard Index) in groundwater samples for adults and children

Heavy metal		Oral exposure						Dermal exposure					
		Adults			Children			Adults			Children		
		Min	Max	Average	Min	Max	Average	Min	Max	Average	Min	Max	Average
CDI	Ni	0.0003	0.0342	0.0169	0.0004	0.0476	0.0235	0.0000	0.0007	0.0004	0.0000	0.0010	0.0005
	Cd	0.0003	0.0034	0.0015	0.0004	0.0047	0.0022	0.0000	0.0000	0.0000	0.0000	0.0000	0.0000
	Pb	0.0074	0.0123	0.0093	0.0103	0.0171	0.0129	0.0002	0.0003	0.0002	0.0002	0.0004	0.0003
	Cr	0.0034	0.0052	0.0042	0.0047	0.0073	0.0059	0.0000	0.0001	0.0000	0.0001	0.0001	0.0001
	As	0.0249	0.0369	0.0286	0.0347	0.0514	0.0398	0.0001	0.0002	0.0001	0.0002	0.0003	0.0002
HQ	Ni	0.0154	1.7077	0.8450	0.0214	2.3786	1.1769	0.0012	0.1321	0.0653	0.0017	0.1938	0.0959
	Cd	0.6154	6.7692	3.0929	0.8571	9.4286	4.3080	0.3212	3.5335	1.6145	0.4714	5.1857	2.3694
	Pb	5.2747	8.7912	6.6077	7.3469	12.245	9.2036	0.3671	0.6119	0.4599	0.5388	0.8980	0.6749
	Cr	1.1282	1.7436	1.4039	1.5714	2.4286	1.9555	2.3557	3.6406	2.9314	3.4571	5.3429	4.3020
	As	83.087	123.08	95.331	115.71	171.43	132.78	1.0577	1.5670	1.2137	1.5523	2.2997	1.7812
HI		90.126	135.71	107.28	125.53	189.02	149.43	4.1041	8.8464	6.2849	6.0231	12.9826	9.2235

**Table 5:** Ratios of carcinogenic effects (*CDI* Chronic Daily Intake, *CR* Cancer Risk, *TCR* Total Carcinogenic Risk) in groundwater samples for adults and children

Heavy metal		Adults			Children		
		Min	Max	Average	Min	Max	Average
CDI	Ni	0.0001	0.0146	0.0072	0.0002	0.0238	0.0118
	Cd	0.0001	0.0015	0.0007	0.0002	0.0024	0.0011
	Pb	0.0032	0.0053	0.0040	0.0051	0.0086	0.0064
	Cr	0.0015	0.0022	0.0018	0.0024	0.0036	0.0029
	As	0.0107	0.0158	0.0123	0.0174	0.0257	0.0199
CR	Ni	0.0001	0.0133	0.0066	0.0002	0.0216	0.0107
	Cd	0.0020	0.0218	0.0099	0.0032	0.0354	0.0162
	Pb	0.00003	0.00004	0.00003	0.00004	0.0001	0.0001
	Cr	0.0006	0.0009	0.0008	0.0010	0.0015	0.0012
	As	0.0160	0.0237	0.0184	0.0260	0.0386	0.0299
TCR		0.0189	0.0556	0.0357	0.0307	0.0903	0.0580

## Results and discussion

### Hydrochemical and physicochemical characteristics

The measured groundwater parameters, including pH, electrical conductivity (EC), temperature, major cations, major anions, and heavy metals, are summarized in Table 3. Their comparison with WHO (2022) guidelines is provided, with separate graphical representations for the two study sites in Figs. 5, 6 and Appendices 2, 3. The pH measurements exhibited a range from 6.8 to 7.5, suggesting a tendency toward neutrality in the analyzed water samples. Electrical conductivity (EC) is a key parameter used to validate physicochemical analyses of water, as it reflects the total concentration of dissolved salts or ionic content in the sample (Bouaissa et al., 2022; Boukich et al., 2024a, 2024b). This parameter varies depending on the concentration and mobility of ions in the solution, both of which are influenced by temperature (Ben-tahar et al., 2025b). The EC values varied significantly, oscillating between 382 µS/cm, indicative of slightly mineralized water, and 873 µS/cm, representative of highly mineralized water (Asmoay et al., 2024; Detay, 1997). The total dissolved solids (TDS) concentrations in the samples ranged from 210 to 480 ppm, classifying them as freshwater (Asmoay et al., 2024; Freeze & Cherry, 1979). Total hardness (TH) readings fluctuated between 113 ppm, categorized as medium hardness, and 187 ppm, denoting hardness (Asmoay et al., 2024; Sawyer et al., 2003). In terms of calcium (Ca^2+^) concentration, values ranged from 25 to 52 ppm, magnesium (Mg^2+^) ranged from 11 to 15 ppm, sodium (Na^+^) values varied between 22 to 35 ppm, and potassium (K^+^) content fluctuated between 1 and 2 ppm. The anionic composition consisted of bicarbonate (HCO₃⁻) concentrations ranging from 120 to 185 ppm, chloride (Cl⁻) levels between 38 and 55 ppm, sulfate (SO₄^2^⁻) values from 29 to 50 ppm, and nitrate (NO₃⁻) readings varying between 1.8 and 4.7 ppm. Phosphate (PO_4_^3−^) concentrations varied from 0.1 to 0.2 ppm, indicating a slight presence of fertilizers (Asmoay et al., 2024). The silica (SiO_2_) content ranged from 17 to 22 ppm in the water samples. All parameters adhered to the guidelines of WHO (2022), suggesting that waters are deemed safe for human consumption. The concentrations of heavy metals included nickel ranging from 0 to 1.1 ppm, cadmium from 0 to 0.1 ppm, lead from 0.2 to 0.4 ppm, chromium from 0.1 to 0.2 ppm, and arsenic ranging from 0.8 to 1.2 ppm. The detected concentrations of heavy metals significantly exceeded the safety thresholds established by the WHO, highlighting serious potential health risk for the local population and rendering the groundwater unsuitable for human consumption (Alhagri et al., 2024). Notably, the average concentrations of heavy metals in the study area were relatively high—comparable to levels reported in other polluted regions such as India (Nayak & Nandimandalam, 2023), Pakistan (Khalid et al., 2020), China (Han et al., 2023), Iran (Rezaei et al., 2019), and Morocco (Gueddari et al., 2022), providing critical insight into the severity of groundwater contamination and highlighting the urgent need for targeted mitigation strategies. In response, a detailed human health risk assessment was undertaken to quantify the adverse effects associated with exposure to these contaminants.

### Groundwater classification

**Fig. 7:**
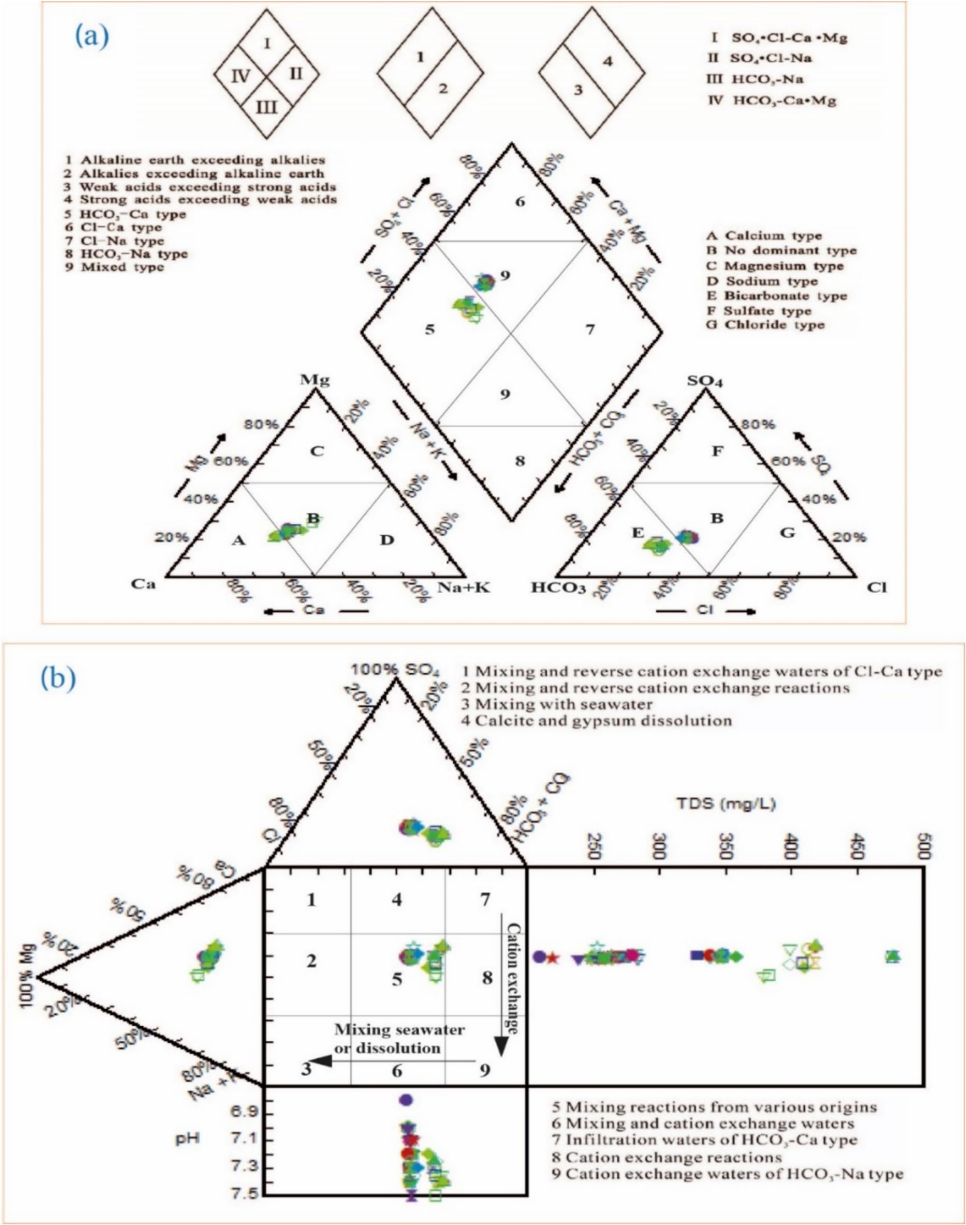
**a**: Piper plot depicting the water type/facies of groundwater samples collected at Wadi Ranyah. **b**: Durov plot showing the classification of these groundwater samples

The Piper plot (Piper, 1944; Xu et al., 2019) of the studied groundwater samples at Wadi Ranyah (Fig. 7 and Table 3) illustrated that the concentration of alkaline earth elements (Ca + Mg) surpassed that of alkali ions (Na + K), while the concentration of the weak acid HCO_3_ was greater than that of the strong acid Cl + SO_4_ in most of the samples, which were located in fields no. 1 and 3. Also, the residual part samples, which were observed in field no. 4, indicated an elevated concentration of strong acids compared to weak acids. This analysis indicated that the two distinct types of water present in the samples were SO_4_.Cl–Ca.Mg and HCO_3_.Ca.Mg. On the other hand, the Durov diagram (Durov, 1948; Xu et al., 2019) demonstrated that the studied samples positioned within field no. 5 (Fig. 7) suggest a mixing reaction arising from diverse sources, characterized by the cation exchange of alkaline earth elements (Ca + Mg) with alkali elements (Na + K). These findings indicated that the water exhibited HCO_3_.Ca.Mg due to the process of ion exchange (Asmoay et al., 2024). Conversely, water samples characterized by SO_4_.Cl-Ca.Mg reflected the influence of evaporite mineral dissolution as a result of interaction (Asmoay et al., 2024).

### Groundwater chemistry

**Fig. 8:**
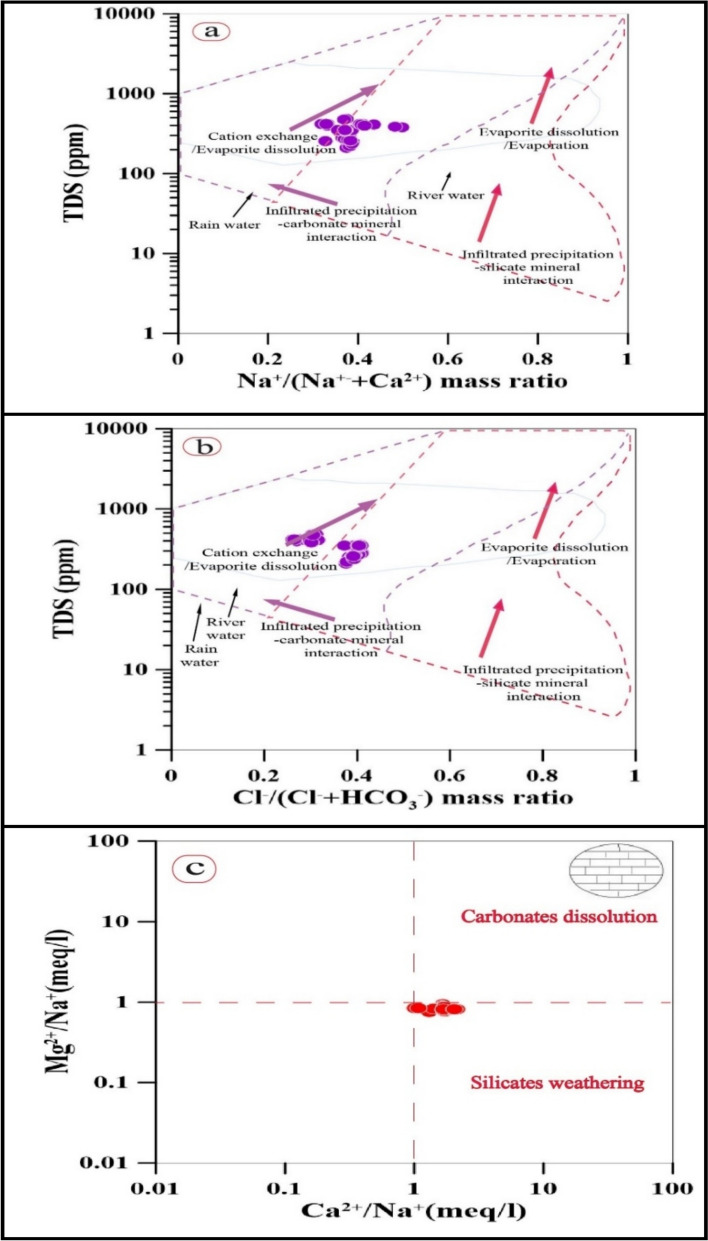
**a** and **b**: Gibbs diagrams illustrating the hydrochemical processes influencing samples. **c**: Bivariate graph of Ca/Na vs. Mg/Na

The Gibbs diagram (Gibbs, 1967; Marandi & Shand, 2018; Ning et al., 2024) for the studied groundwater samples (Fig. 8) illustrated that the predominant mechanisms governing the water chemistry are cation exchange and the dissolution of evaporite minerals. A bivariate plot comparing the Ca^2+^/Na^+^ ratio to the Mg^2+^/Na^+^ ratio indicated that silicate weathering plays a role in shaping water chemistry (Asmoay & Mabrouk, 2024). To conclude, the interactions among the dissolution of evaporites, cation exchanges, and silicate weathering have significantly affected the water chemistry of the examined samples. Furthermore, this has resulted in the presence of two distinct water types and an elevated concentration of alkaline earth elements (Ca^2+^, Mg^2+^), alongside weak acids such as HCO_3_ and alkali ions (Na^+^, K^+^), as well as strong acids like SO_4_^2−^ and Cl^−^.

### Non-carcinogenic impact of heavy metal content

Non-carcinogenic effects exert their influence on humans through two distinct pathways, the ingestion or oral route, and the dermal contact. The Hazard Quotient (HQ) and Hazard Index (HI) values delineate the extent of the impact of water consumption on residents, necessitating that these values surpass the threshold of unity as stipulated by the US EPA (2014).

#### Oral exposure

**Fig. 9:**
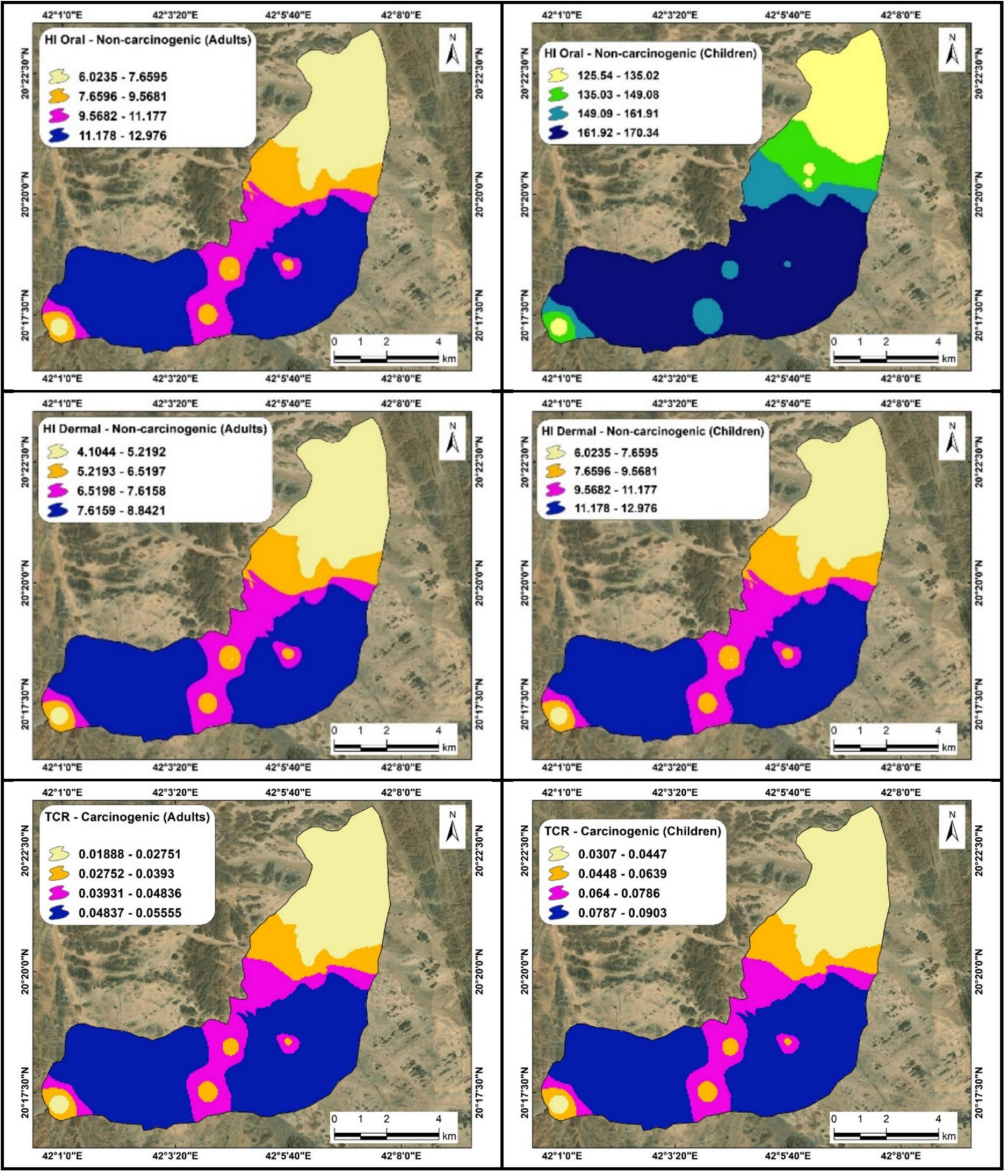
Distribution maps of HI (oral) and HI (dermal) illustrating non-carcinogenic effects for adults and children, along with TCR maps representing carcinogenic effects for both age groups, at site number 1

**Fig. 10:**
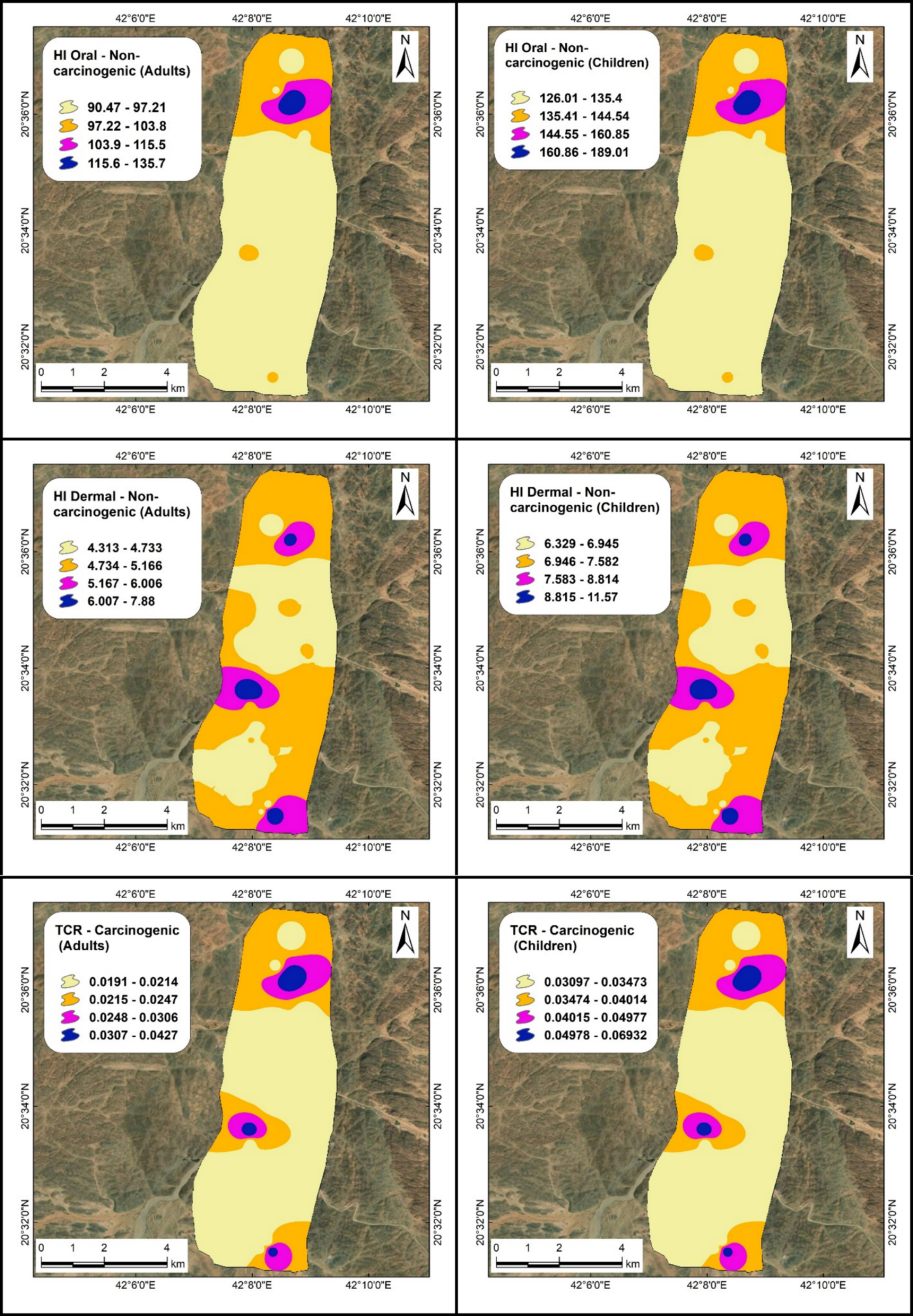
Distribution maps of HI (oral) and HI (dermal) illustrating non-carcinogenic effects for adults and children, along with TCR maps representing carcinogenic effects for both age groups, at site number 2

In this study, the HQ values resulting from oral exposure (Figs. 9, 10 and Table 4) to five heavy metals for adults, based on their mean values, can be ranked in descending order as follows: As (95.0) > Pb (6.6) > Cd (3.0) > Cr (1.4) > Ni (0.8), with the HI mean value of 107. Conversely, for children, the values are ranked as: As (132.0) > Pb (9.0) > Cd (4.0) > Cr (2.0) > Ni (1.2), with an HI mean value of 149. The mean HQ value for Ni does not surpass the US EPA threshold of unity, indicating a lesser impact for adults, whereas it exceeds the safe limit in the case of children, potentially leading to increased effects. Arsenic concentration in water samples poses a greater risk to children than to adults, followed by lead, cadmium, chromium, and a least impact from nickel.

#### Dermal exposure

The HQ values derived from dermal exposure in adults (Figs. 9, 10 and Table 4), calculated based on their average values, can be organized in descending order as follows: Cr (3.0) > Cd (1.6) > As (1.2) > Pb (0.4) > Ni (0.1), with an HI mean value of 107. For children, the values are classified as: Cr (4.0) > Cd (2.4) > As (1.8) > Pb (0.7) > Ni (0.1), with an HI of 9. The average HQ values for lead and nickel are below the safe limit, indicating no health risk for either adults or children. The chromium concentration in water samples has a more pronounced effect on children than on adults, followed by cadmium, arsenic, and chromium.

To conclude, arsenic emerged as the most significant contaminant in groundwater samples due to oral exposure for both adults and children, whereas nickel exhibit the least impact. In terms of dermal exposure, chromium had the highest effect on both adults and children, surpassing cadmium and arsenic, while lead and nickel posed no significant threat. The risk attributable to oral exposure constitutes 95% of the overall hazard when contrasted with dermal exposure, which accounts for 5%. On the whole, the non-carcinogenic effects are more pronounced in children than in adults. These non-carcinogenic effects identified in the groundwater of Wadi Ranyah are notably more severe compared to those reported in other countries such as Egypt, Qatar, and Morocco (Asmoay et al., 2024; Manawi et al., 2024; Sanad et al., 2024). These findings are also consistent with researches conducted in other regions of Saudi Arabia (Alhagri et al., 2024; Alharbi & El-Sorogy, 2024; Ali et al., 2020; Khan et al., 2023). This comparative analysis was conducted to underscore regional disparities in groundwater quality and the corresponding health risk, highlighting the urgent need for localized mitigation strategies tailored to Wadi Ranyah’s specific environmental and anthropogenic conditions.

The non-carcinogenic effects of cadmium on human health include renal impairment and bone demineralization (Belle et al., 2024), while arsenic exposure is associated with dermatological lesions, cardiovascular disorders, and neurological repercussions (Niknejad et al., 2023). Chromium exposure may result in dermal irritation, ulceration, and respiratory complications (Zhang et al., 2023). Lead exposure is particularly detrimental to pediatric populations, leading to developmental delays, cognitive impairments, and neurological harm. In adult populations, it is linked to hypertension and renal dysfunction (Moradnia et al., 2024). Nickel exposure is associated with contact dermatitis, respiratory ailments, and hypersensitivity reactions (Belle et al., 2024).

### Carcinogenic impact of heavy metal content

Carcinogenic consequences can impact an individual through ingestion; cancer risk (CR) values illustrate the influence of water intake on local inhabitants, necessitating that these metrics exceed the benchmark of 10^–6^ as designated by the US EPA (2014). Our findings (Figs. 9, 10 and Table 5) related to cancer risk from dermal exposure among adults, derived from mean values, can be ranked in descending order as follows: As (0.018) > Cd (0.009) > Ni (0.006) > Cr (0.0008) > Pb (0.00003), yielding a mean total cancer risk (TCR) of 0.0357. For children, the values are as follows: As (0.02) > Cd (0.016) > Ni (0.011) > Cr (0.0012) > Pb (0.0001), resulting in a mean TCR of 0.0580. The concentration of arsenic in water samples presented a higher cancer risk for children compared to adults, followed by cadmium, nickel, chromium, and lead, which exert the least influence.

The diseases attributable to the carcinogenic effects of cadmium include an elevated risk of lung cancer and associations with prostate and renal cancers (Khoshakhlagh et al., 2024), while arsenic is linked to skin cancer as well as malignancies of the bladder, lungs, and liver (Coradduzza et al., 2024). Chromium exposure is implicated in lung cancer and has associations with cancers of the nasal cavity and sinuses (Ngole-Jeme & Fantke, 2017). Lead exposure is associated with an increased risk of lung, gastric, and brain cancers (Cai et al., 2019). Nickel exposure is linked to lung and nasal cancers (Khoshakhlagh et al., 2024).

## Conclusions

The inhabitants of Wadi Ranyah, Saudi Arabia, predominantly rely on groundwater as their primary water source. Physicochemical parameters, including pH, EC, TDS, and TH, along with PO_4_^3^⁻ and SiO_2_, were mostly within WHO guidelines, indicating generally acceptable quality in terms of major constituents. However, the water exhibited moderate to high mineralization and hardness, and hydrochemical analysis revealed two dominant water types: SO_4_·Cl–Ca·Mg and HCO_3_–Ca·Mg. These were shaped by processes such as ion exchange, evaporite dissolution, and silicate weathering, as evidenced by Piper, Durov, Gibbs, and bivariate diagrams.

Critically, heavy metals—particularly arsenic, lead, cadmium, chromium, and nickel—exceeded WHO and US EPA safety thresholds. Health risk assessment indicated that oral exposure posed the most significant non-carcinogenic and carcinogenic risks, especially for children, with arsenic and lead as the primary contributors. Dermal exposure risk was lower but still non-negligible, particularly due to chromium and cadmium. These exposures may lead to severe health consequences, including dermatological, cardiovascular, neurological disorders, and increased cancer risks (e.g., lung, renal, and gastric cancers).

Given these findings, the groundwater in Wadi Ranyah is currently unsuitable for direct human consumption. To mitigate this health risk, the implementation of advanced filtration technologies—such as reverse osmosis and ion exchange—is strongly recommended. In addition, regular monitoring and sustainable management practices will be essential to ensure long-term water safety and protect public health.

## Data Availability

No datasets were generated or analysed during the current study.

## Appendix 1

Physico-chemical characteristics of groundwater samples in Wadi Ranyah. Temperature (T) in °C, pH, electrical conductivity (EC) expressed in μS/cm, while total dissolved solids (TDS), total hardness (TH), major cations, major anions, and heavy metals are expressed in ppm.

**Table:**

Well no	T	pH	EC	TDS	TH	Ca^2+^	Mg^2+^	Na^+^	K^+^	HCO_3_^−^	Cl^−^	SO_4_^2−^	NO_3_^−^	PO_4_^3−^	SiO_2_	Ni	Cd	Pb	Cr	As
1	24.0	7.30	500	275	155	42.0	12.3	28.5	1.5	128	50.2	42.0	2.5	0.10	18.6	0.03	0.01	0.25	0.12	0.81
2	22.0	7.10	455	250	133	35.0	11.2	25.4	1.2	124	45.6	39.0	2.3	0.22	22.1	1.08	0.10	0.27	0.15	1.03
3	23.0	7.20	482	265	148	40.0	11.8	27.6	1.4	126	48.5	40.0	2.4	0.23	22.2	1.05	0.11	0.28	0.16	1.02
4	22.0	7.10	473	260	142	38.0	11.5	26.8	1.3	125	47.2	39.0	2.3	0.21	22.3	1.10	0.09	0.26	0.14	1.04
5	25.0	7.40	509	280	162	44.0	12.6	29.7	1.6	130	52.3	43.0	2.6	0.22	22.1	1.09	0.10	0.27	0.15	1.03
6	21.0	7.00	464	255	138	37.0	11.2	26.2	1.2	124	46.1	38.0	2.2	0.22	22.1	1.08	0.10	0.27	0.15	1.03
7	23.0	7.30	491	270	152	41.0	12.0	28.0	1.4	127	49.4	41.0	2.5	0.22	22.1	1.08	0.10	0.27	0.15	1.03
8	22.0	7.20	482	265	145	39.0	11.6	27.4	1.3	126	47.8	40.0	2.4	0.22	22.1	1.08	0.10	0.27	0.15	1.03
9	25.0	7.40	518	285	165	45.0	12.8	30.1	1.6	131	52.9	44.0	2.6	0.22	22.1	1.08	0.10	0.27	0.15	1.03
10	21.0	7.00	455	250	136	36.0	11.1	25.3	1.2	123	45.5	38.0	2.2	0.22	22.1	1.08	0.10	0.27	0.15	1.03
11	23.0	7.20	482	265	148	40.0	11.8	27.6	1.4	126	48.5	40.0	2.4	0.08	19.0	0.89	0.01	0.29	0.17	0.98
12	22.0	7.10	473	260	142	38.0	11.5	26.8	1.3	125	47.2	39.0	2.3	0.10	18.0	0.85	0.01	0.28	0.14	1.02
13	25.0	7.40	509	280	162	44.0	12.6	29.7	1.6	130	52.3	43.0	2.6	0.22	22.1	1.08	0.10	0.27	0.15	1.03
14	21.0	7.00	464	255	138	37.0	11.2	26.2	1.2	124	46.1	38.0	2.2	0.22	22.2	1.09	0.10	0.28	0.15	1.03
15	23.0	7.30	491	270	152	41.0	12.0	28.0	1.4	127	49.4	41.0	2.5	0.22	22.1	1.08	0.10	0.27	0.15	1.03
16	22.0	7.20	482	265	145	39.0	11.6	27.4	1.3	126	47.8	40.0	2.4	0.22	22.1	1.08	0.10	0.27	0.15	1.03
17	22.0	7.20	482	265	145	39.0	11.6	27.4	1.3	126	47.8	40.0	2.4	0.22	22.1	1.08	0.10	0.27	0.15	1.03
18	22.0	7.10	455	250	133	35.0	11.2	25.5	1.2	124	45.1	39.0	2.3	0.22	22.1	1.08	0.10	0.27	0.15	1.03
19	23.0	7.20	462	254	155	44.0	10.9	24.6	1.3	128	46.2	44.0	2.1	0.22	22.1	1.08	0.10	0.27	0.15	1.03
20	21.0	7.00	464	255	139	37.0	11.3	26.3	1.2	124	46.3	38.0	2.2	0.22	22.1	1.08	0.10	0.27	0.15	1.03
21	24.0	7.30	504	277	159	43.0	12.5	29.5	1.5	129	51.5	42.0	2.5	0.22	22.1	1.08	0.10	0.27	0.15	1.03
22	22.0	7.20	482	265	145	39.0	11.6	27.4	1.3	126	47.9	40.0	2.4	0.20	17.9	1.02	0.01	0.31	0.14	1.02
23	21.0	7.00	455	250	136	36.0	11.1	25.4	1.2	123	45.6	38.0	2.2	0.23	22.2	1.05	0.11	0.28	0.16	1.02
24	24.0	7.30	500	275	155	42.0	12.3	28.5	1.5	128	50.3	42.0	2.5	0.21	22.3	1.10	0.09	0.26	0.14	1.04
25	22.0	7.10	473	260	142	38.0	11.5	26.9	1.3	125	47.3	39.0	2.3	0.22	22.1	1.09	0.10	0.27	0.15	1.03
26	25.0	7.40	509	280	164	45.0	12.6	29.8	1.6	130	52.4	43.0	2.6	0.22	22.2	1.10	0.10	0.28	0.15	1.03
27	21.0	7.00	464	255	138	37.0	11.2	26.3	1.2	124	46.2	38.0	2.2	0.23	22.2	1.06	0.11	0.28	0.16	1.02
28	23.0	7.30	491	270	152	41.0	12.0	28.0	1.4	127	49.5	41.0	2.5	0.21	22.3	1.11	0.09	0.26	0.14	1.04
29	22.0	7.20	482	265	145	39.0	11.6	27.5	1.3	126	47.9	40.0	2.4	0.22	22.1	1.09	0.10	0.27	0.15	1.03
30	25.0	7.40	509	280	162	44.0	12.6	29.8	1.6	130	52.4	43.0	2.6	0.22	22.2	1.10	0.10	0.28	0.15	1.03
31	21.0	7.00	455	250	136	36.0	11.1	25.3	1.2	123	45.5	38.0	2.2	0.23	22.2	1.05	0.11	0.28	0.16	1.02
32	23.0	7.20	482	265	148	40.0	11.8	27.6	1.4	126	48.6	40.0	2.4	0.21	22.3	1.10	0.09	0.26	0.14	1.04
33	22.0	7.10	518	285	142	38.0	11.5	26.9	1.3	125	47.3	39.0	2.3	0.22	22.1	1.09	0.10	0.27	0.15	1.03
34	28.0	7.50	636	350	187	50.0	15.0	35.0	2.0	140	55.0	50.0	3.0	0.23	22.2	1.06	0.11	0.28	0.16	1.02
35	19.0	6.80	382	210	125	32.0	11.0	22.0	1.0	120	42.0	32.0	1.8	0.21	22.3	1.11	0.09	0.26	0.14	1.04
36	27.0	7.40	618	340	177	48.0	14.0	33.0	1.9	135	53.0	48.0	2.9	0.22	22.1	1.09	0.10	0.27	0.15	1.03
37	20.0	7.10	400	220	132	34.0	11.5	24.0	1.1	125	44.0	34.0	2.1	0.12	18.0	0.96	0.01	0.27	0.15	1.10
38	26.0	7.30	600	330	170	46.0	13.5	31.0	1.8	130	51.0	46.0	2.8	0.09	17.0	1.00	0.02	0.28	0.14	0.98
39	21.0	7.00	436	240	136	36.0	11.3	26.0	1.2	122	46.0	36.0	2.2	0.18	21.6	1.01	0.02	0.32	0.14	1.01
40	25.0	7.30	509	280	162	44.0	12.8	29.0	1.6	128	49.5	44.0	2.6	0.10	18.6	0.03	0.01	0.25	0.12	0.81
41	22.0	7.20	473	260	143	38.0	11.6	27.0	1.3	126	47.0	38.0	2.4	0.10	18.7	0.03	0.01	0.36	0.12	0.85
42	24.0	7.30	491	270	156	42.0	12.4	28.5	1.5	129	50.5	42.0	2.5	0.11	18.8	0.03	0.01	0.35	0.13	0.82
43	23.0	7.20	482	265	146	39.0	11.9	27.8	1.4	127	48.0	39.0	2.4	0.11	18.8	0.03	0.01	0.35	0.13	0.82
44	23.0	7.20	482	265	149	40.0	11.9	27.8	1.4	127	48.0	40.0	2.4	0.09	18.5	0.02	0.01	0.24	0.11	0.81
45	22.0	7.20	473	260	143	38.0	11.6	27.0	1.3	126	47.0	38.0	2.4	0.10	18.6	0.03	0.01	0.26	0.12	0.82
46	24.0	7.30	491	270	156	42.0	12.4	28.5	1.5	129	50.5	42.0	2.5	0.09	18.5	0.02	0.01	0.24	0.11	0.81
47	23.0	7.20	482	265	146	39.0	11.9	27.8	1.4	127	48.0	39.0	2.4	0.11	18.8	0.03	0.01	0.35	0.13	0.82
48	23.0	7.20	482	265	149	40.0	11.9	27.8	1.4	127	48.0	40.0	2.4	0.09	18.5	0.02	0.01	0.24	0.11	0.81
49	22.0	7.20	473	260	143	38.0	11.6	27.0	1.3	126	47.0	38.0	2.4	0.10	18.6	0.03	0.01	0.26	0.12	0.82
50	25.0	7.20	618	340	145	40.0	11.0	27.0	1.2	120	48.0	40.0	2.2	0.10	19.6	0.01	0.10	0.40	0.14	0.81
51	23.0	7.40	655	360	163	44.0	13.0	30.0	1.8	130	52.0	44.0	2.8	0.10	18.6	0.03	0.01	0.25	0.12	0.81
52	24.0	7.30	627	345	152	41.0	12.0	28.0	1.4	125	49.0	41.0	2.4	0.10	18.7	0.03	0.01	0.36	0.12	0.85
53	24.5	7.35	636	350	159	43.0	12.5	29.0	1.6	127	51.0	43.0	2.6	0.09	19.1	0.02	0.01	0.32	0.15	0.83
54	24.0	7.30	633	348	155	42.0	12.2	28.5	1.5	128	50.0	42.0	2.5	0.09	19.2	0.01	0.01	0.40	0.13	0.82
55	24.0	7.30	633	348	155	42.0	12.3	28.5	1.5	128	50.2	42.0	2.5	0.10	18.8	0.03	0.01	0.30	0.12	0.81
56	26.0	7.40	764	420	175	50.0	12.2	28.1	1.4	185	38.8	38.2	4.6	0.09	18.9	0.03	0.01	0.35	0.11	0.84
57	21.0	7.30	869	478	153	40.2	12.8	27.6	1.6	165.3	41.8	33.8	4.1	0.09	19.2	0.01	0.01	0.40	0.13	0.82
58	24.0	7.30	745	410	138	35.2	12.1	27.8	1.8	178.5	44.0	28.9	4.5	0.10	18.8	0.03	0.01	0.30	0.12	0.81
59	24.0	7.35	636	350	149	39.0	12.5	28.2	1.4	130	48.5	40.0	2.6	0.09	18.9	0.03	0.01	0.35	0.11	0.84
60	26.0	7.40	755	415	173	49.0	12.4	28.0	1.5	180	40.0	38.5	4.5	0.09	19.4	0.02	0.01	0.31	0.14	0.83
61	25.0	7.40	764	420	138	35.0	12.2	27.9	1.6	170	40.0	39.0	3.7	0.09	19.2	0.01	0.01	0.40	0.13	0.82
62	21.0	7.30	873	480	155	41.0	12.9	27.7	1.7	160	42.0	34.0	4.0	0.09	18.9	0.03	0.01	0.35	0.11	0.84
63	24.0	7.30	755	415	140	36.0	12.2	27.9	1.9	180	44.5	30.0	4.4	0.09	19.4	0.02	0.01	0.31	0.14	0.83
64	23.0	7.20	749	412	134	34.0	12.0	30.0	1.9	155	41.5	35.0	3.8	0.09	19.2	0.01	0.01	0.40	0.13	0.82
65	23.5	7.25	636	350	155	41.5	12.5	28.2	1.6	130.5	49.5	40.5	2.8	0.10	19.6	0.01	0.10	0.40	0.14	0.81
66	26.5	7.35	727	400	175	49.5	12.4	28.3	1.5	183	39.2	37.5	4.4	0.10	18.8	0.03	0.01	0.30	0.12	0.81
67	24.5	7.35	745	410	139	35.5	12.2	27.9	1.7	167	39.5	39.0	3.6	0.09	18.9	0.03	0.01	0.35	0.11	0.84
68	22.5	7.40	691	380	113	24.5	12.5	27.9	1.2	176.5	44.2	34.5	4.1	0.09	19.4	0.02	0.01	0.31	0.14	0.83
69	20.5	7.25	873	480	154	40.5	12.9	27.7	1.7	166	42.0	32.5	4.3	0.09	19.2	0.01	0.01	0.40	0.13	0.82
70	24.5	7.25	727	400	136	34.5	12.2	27.9	1.9	180	44.5	29.5	4.7	0.10	18.8	0.03	0.01	0.30	0.12	0.81
71	24.0	7.30	636	350	162	45.0	12.1	28.5	1.5	130	45.0	40.0	2.5	0.09	18.9	0.03	0.01	0.35	0.11	0.84
72	26.0	7.40	764	420	179	52.0	12.0	28.0	1.4	185	38.0	38.0	4.6	0.09	19.4	0.02	0.01	0.31	0.14	0.83
73	23.0	7.5	700	385	115	26.0	12.3	27.8	1.3	175	44.0	35.0	3.9	0.10	18.8	0.03	0.01	0.30	0.12	0.81
74	21.0	7.3	869	478	155	41.0	12.8	27.6	1.6	165	42.0	34.0	4.1	0.09	19.1	0.02	0.01	0.35	0.11	0.85
75	24.0	7.3	633	348	154	42.0	12.0	28.5	1.5	128	50.0	42.0	2.5	0.21	20.5	1.01	0.03	0.35	0.15	1.2
76	24.0	7.3	633	348	155	42.0	12.3	28.5	1.5	128	50.2	42.0	2.5	0.09	18.9	0.03	0.01	0.35	0.11	0.84
77	26.0	7.4	764	420	175	50.0	12.2	28.1	1.4	185	38.8	38.2	4.6	0.09	19.4	0.02	0.01	0.31	0.14	0.83

## Appendix 2

Distribution maps of selected parameters in groundwater samples for site number 1. EC is expressed in μS/cm, while TH, major cations and major anions are shown in ppm.

## Appendix 3

Distribution maps of selected parameters in groundwater samples for site number 2. EC is expressed in μS/cm, while TH, major cations and major anions are shown in ppm.
